# Extrafoveal Processing in Categorical Search for Geometric Shapes: General Tendencies and Individual Variations

**DOI:** 10.1111/cogs.13025

**Published:** 2021-08-11

**Authors:** Anna Dreneva, Anna Shvarts, Dmitry Chumachenko, Anatoly Krichevets

**Affiliations:** ^1^ Faculty of Psychology Lomonosov Moscow State University; ^2^ Freudenthal Institute Faculty of Science Utrecht University

**Keywords:** Extrafoveal processing, Categorical search, Geometric shapes, Individual differences, Covert attention

## Abstract

The paper addresses the capabilities and limitations of extrafoveal processing during a categorical visual search. Previous research has established that a target could be identified from the very first or without any saccade, suggesting that extrafoveal perception is necessarily involved. However, the limits in complexity defining the processed information are still not clear. We performed four experiments with a gradual increase of stimuli complexity to determine the role of extrafoveal processing in searching for the categorically defined geometric shape. The series of experiments demonstrated a significant role of extrafoveal processing while searching for simple two‐dimensional shapes and its gradual decrease in a condition with more complicated three‐dimensional shapes. The factors of objects’ spatial orientation and distractor homogeneity significantly influenced both reaction time and the number of saccades required to identify a categorically defined target. An analysis of the individual *p*‐value distributions revealed pronounced individual differences in using extrafoveal analysis and allowed examination of the performance of each particular participant. The condition with the forced prohibition of eye movements enabled us to investigate the efficacy of covert attention in the condition with complicated shapes. Our results indicate that both foveal and extrafoveal processing are simultaneously involved during a categorical search, and the specificity of their interaction is determined by the spatial orientation of objects, type of distractors, the prohibition to use overt attention, and individual characteristics of the participants.

## 1 Introduction

Categorizing and identifying stimuli out of the fovea poses a challenge in the field of visual search investigation. Although perceptual mechanisms have been studied for a long time, there still exist a lot of theoretical controversies, little interpretation consensus, and a number of methodological challenges. These limit the possible answers to the question of what can be processed without foveal attention under different conditions. Moreover, in many situations, we cannot unambiguously ponder what is perceived without foveal vision, given the variety of objects that could be classified into different classes in terms of their complexity for perception. Clearly, some information is easier to identify without thorough foveal analysis, while processing other information requires the help of a direct gaze.

While researchers continue to argue over the features that can be considered high level, they agree on those that are broadly accepted as low level—including color, shape, spatial orientation, and motion (Wolfe, 2018; Wolfe & Horowitz, 2017). The low‐level features for selecting relevant information were given a central role in feature‐integration theory, proposed by Treisman and Gelade (1980). It assumes two stages in the work of attention, being closely connected and working one after another. During the first stage, visual features are processed in parallel in a pre‐attentive, independent, and simultaneous manner; that is, in a bottom‐up fashion. During the second stage, the most relevant features defined at the previous stage are studied in a serial manner with overt attention. A similar idea was suggested by Wolfe in his guided search model, who proposed that visual features guide attention in both bottom‐up and top‐down manners (Wolfe, 1994).

It is not only low‐level visual features that can guide overt attention. Semantic information about an object category, through a word label, can control overt attention in a top‐down fashion (e.g., Chen & Zelinsky, 2006; Malcolm & Henderson, 2010; Zelinsky, 2008; Zelinsky, Adeli, Peng, & Samaras, 2013). Moreover, recent research shows that this semantic guidance may happen outside the fovea (Cimminella, Della Sala, & Coco, 2020; Strasburger, Rentschler, & Jüttner, 2011).

Traditionally, the visual field is divided into three regions extending from the retina center to the periphery: (i) the fovea, covering a visual angle of 1° eccentricity and providing a vision of high resolution; (ii) the parafovea, stretching out to 4–5° of eccentricity; and (iii) the periphery, embracing the rest of the visual field (Larson & Loschky, 2009). For the purposes of this work, extrafoveal vision will be defined as the processing of information appearing in the parafovea or periphery. Despite a pronounced decrease of visual acuity outside the fovea, different sorts of higher‐level information can be accrued in extrafoveal vision (Cimminella et al., 2020) as soon as the stimuli appear (Auckland, Cave, & Donnelly, 2007).

The ability to identify semantic information and guide early overt attention within extrafoveal vision contradicts the principles of the traditional feature‐integration theory (Treisman & Gelade, 1980) as well as its more recent updates (Evans & Treisman, 2005; Treisman, 2006). In recent work, Cimminella et al. (2020) checked the contribution of extrafoveal vision to searching for stimuli in real‐world scenes and revealed that object semantics can be processed extrafoveally and then used to guide saccadic programming in a top‐down manner. Their findings contribute to the debate concerning the role of semantic information on eye movement guidance and open up perspectives for investigating extrafoveal processing while searching for various kinds of stimuli, including complex ones.

One of the methodological challenges in the research of extrafoveal processing has been the difficulty in empirically dissociating between pre‐attentive processing and covert attention, both of which participate in preparing eye movements and thus guiding overt attention. It is reasonable to consider pre‐attentive processing as a specific type of processing that functions before selective attention is guided to a particular object (Wolfe, 2018) and that detects something visible and identifiable before secective attention has been applied (Wolfe & Utochkin, 2019). Moreover, in some models, pre‐attentive and attentive types of search are considered to be the same process differing only in the degree of spreading attention. For example, in Treisman and Gormican (1988), pre‐attentive search is defined as a “search in which attention is distributed widely over the whole display” (p. 43). After the object of interest has been chosen, selective attention can be attracted to it either overtly (with eye movements) or covertly (without eye movements; Posner, 1980, 2016). Then, the visual field is investigated through all types of attention according to the task. In this situation, when both pre‐attentive processing and covert attention behave tacitly, invisibly for the researcher, their clear dissociation from each other presents a tricky methodological issue that cannot be settled within our study. Therefore, in the current work, we use the term “extrafoveal processing,” which comprises both types of covert processing for identifying features or objects outside the fovea region before their analysis by overt attention begins.

### 1.1 Categorical search

While a lot of research concerns the question of how attention is guided by visual features (i.e., direct targeting), we focus on semantic information (i.e., indirect targeting) and aim to identify the limit of extrafoveal processing. The latter type of search is called categorical search. In this case, the target object is defined only by its category, so an observer does not know its exact features. Searching for some object when we know only its name and presuppose some typical features is quite common and ecologically relevant. Although this is the kind of search we most naturally exploit in our daily lives, it is not as studied as the visual feature‐based search due to obvious complexities in organizing experiments. Still, categorical search has been examined for the past 50 years.

Investigations of categorical search date back to a pioneering study by Jonides and Gleitman (1972), who highlighted the importance of categorical search and the influence of feature similarity relationships on categorical search effects. Subsequent research efforts tried to distinguish between the roles of a category with a distinct pool of semantic information and visual features (Dahan & Tanenhaus, 2005). They revealed that observers tended to fixate on distractors that resembled typical members of the target category; for example, while looking for a snake they fixated on a rope. Yet this preference turned out to be asymmetrical: While looking for a rope, the participants hardly preferred to look at the snake. This asymmetry indicated that the search was guided by the target category and not by the visual similarity to an exact template.

Despite numerous findings that searching for pictorially previewed targets is much more efficient as compared to a text label (e.g., Vickery, King, & Jiang, 2005; Wolfe, Horowitz, Kenner, Hyle, & Vasan, 2004), in real life, we often do not know exactly what an object looks like. Still, some studies report an above‐chance guidance of eye movements to categorically defined targets (Yang & Zelinsky, 2006, 2009; Zelinsky et al., 2013). For instance, Yang and Zelinsky (2009) compared the target preview and categorical conditions and showed that although the observers in the categorical condition had been looking for teddy bear targets longer and made more eye movements, they still fixated targets far sooner than would have been expected in the case of fixating the objects randomly. This study indicated that such guidance uses a categorical model that consists of specific features common to the target category. Another study (Schmidt & Zelinsky, 2009) demonstrated that the guidance was gradually proportional to the information available from the target cue. These results confirm that complex objects can be effectively identified through their category in extrafoveal vision.

Research using different kinds of complex stimuli (e.g., real‐world scenes) in a dual‐task paradigm has demonstrated no decrement in identifying a vehicle or an animal shown in the periphery while performing a challenging task at fixation (F. F. Li, VanRullen, Koch, & Perona, 2002). These results indicate that high‐level object representations may be accessed in a parallel manner, providing efficient categorization and identification even outside the focus of attention (Greene & Fei‐Fei, 2014; Koch & Tsuchiya, 2007; Poncet, Reddy, & Fabre‐Thorpe, 2012). Due to its high ecological validity, a categorical search task is relevant to studying visual attention while looking for complex stimuli of different categories that also include scientific categories and objects.

### 1.2 Why geometric shapes

Geometric shapes presenting mathematical concepts correspond to a great variety of categories with numerous members that can appear under different angles. Some of the first eye movement experiments on visual search for simple geometric shapes were conducted by Williams (1966). He measured search times in a number of conditions varying the features of target objects, such as color, shape, and size. He found that color speeded up the search process substantially, while the specification of size turned out to be much less effective, and the shape specification provided almost no advantage. Later, these results appeared to be task‐specific as other studies (Gould & Dill, 1969; Viviani & Swensson, 1982) revealed the ability to use shape information to guide eye movements. However, the above‐mentioned research also reported that distractors were also scanned, while Williams (1966) demonstrated that saccadic eye movements could be made directly to the targets, thus revealing the capability of extrafoveal processing.

In the context of investigating extrafoveal search limitations, geometric shapes can be useful to manipulate the degree of difficulty, which is notably different in the case of searching for a circle versus for a six‐angled pyramid, revealing apparent differences in the shapes’ complexity, and identifying 2‐dimentional (2‐D) versus identifying 3‐dimentional (3‐D) stimuli. These differences have been shown by Pilon and Friedman (1998) who found that the stimulus complexity itself, rather than the depicted dimensionality, could explain different performances in 2‐D and 3‐D conditions. In contrast, the work of Brown, Weisstein, and May (1992) provided evidence that simple 3‐D shapes were not processed in parallel and showed independent pathways during early visual processing. Moreover, attentional processing was definitely required to differentiate one shape from another. Still, some 3‐D shapes used in their study had such dissimilar 2‐D features that they always popped out from each other. So visual search appeared to be based only on 2‐D featural differences, and the authors could not identify the relative contribution of 2‐D and 3‐D form differences from these results. However, it is important to differentiate these types of conditions, as they might present different levels of stimuli complexity.

Pilon and Friedman (1998) also demonstrated a pronounced influence of practice with 2‐D displays on the following identification of the quasi‐3‐D shapes. The detected role of training allowed the authors to conclude that the mechanisms underlying pre‐attentive processing might differ from the mechanisms of simple training of automatic processing. In the context of mathematical concepts, it may indicate that experts in this area can expose specific patterns of attention and search because the initially high‐level operations begin to function automatically and influence low‐level behavior including preparing and executing saccades. Meanwhile, the question of whether new pre‐attentive features can be learned through practice is still unresolved. The pertinent literature on the subject (Carrasco, Ponte, Rechea, & Sampedro, 1998; Schneider & Shiffrin, 1977) provides only fractional data that previously the inefficient conjunction (Ellisson & Walsh, 1998; Lobley & Walsh, 1998) or even categorical (F. F. Li et al., 2002; Treisman, 2006) searches can be made more efficient with training. The issue of expert abilities is closely connected to inter‐individual variability and individual perceptual strategies, which should also be considered while investigating extrafoveal processing.

### 1.3 The purpose of the current study

Taken together, there are still a number of open questions concerning the capabilities of extrafoveal processing during the categorical search. First, the difficulty level of objects that necessarily requires overt attention remains unclear. Second, even though a lot of research has revealed general attentional mechanisms, all individuals tend to utilize their own perception strategies, which seem to be minimally investigated. Third, extrafoveal processing often takes some time before passing the baton to foveal attention, so the capability of the extrafoveal search when eye movements are prohibited presents a worthy research question.

The current study aims to examine the capabilities of extrafoveal processing while searching for geometric shapes of different levels of difficulty. In the series of experiments, we used geometric shapes, which become more and more complicated with each experiment, varying from simple geometric shapes (a cross or a circle) to very difficult 3‐D shapes (three‐ to six‐angled pyramids). We aimed to test the capabilities of early pre‐attentive processing of categorically defined stimuli and document the specific level of the shapes’ difficulty that makes it impossible for the participants to identify the target shape without overt attention.

The present study used a novel methodological approach based on the works of Zelinsky et al. (2006, 2009, 2011, 2013), who used the percentage of trials when the very first saccade landed on the target. We modified this parameter and calculated a more sensitive indicator considering the order number of the target area visit, which was then compared with the random search value. This parameter allowed us to investigate extrafoveal processing that detects the most likely location of a target object.

Although the general tendencies of interaction between attention and eye movements during a categorical search for geometric shapes are quite intriguing, here, we also pay attention to individual differences and search strategies, which vary widely across individuals. We used a novel statistical method that allowed us to analyze not only the mean performance values of the whole sample but individual measures of search efficacy as well.

In light of the above‐mentioned knowledge gaps, we ran a series of experiments and analyzed under‐investigated categorical processing, while searching for a geometric shape of various difficulty. The purpose of the study was therefore threefold: (i) to investigate the dependence between the efficacy of extrafoveal processing and the degree of stimulus difficulty while searching for a categorically defined geometric shape; (ii) to describe individual search strategies and the degree of pre‐attentive, extrafoveal processing involvement during categorical search; and (iii) to study the plasticity of extrafoveal processing by triggering it in an experiment that forced the prohibition of eye movements.

Experiment 1 served as a pilot study investigating pre‐attentive, extrafoveal processing in the condition of simple geometric shapes such as a circle, a square, a triangle, and a cross. Experiment 2 included more complicated shapes such as rectangles and squares among homogeneous or heterogeneous distractors and two spatial orientations for all shapes. Experiment 3 involved even harder 3‐D stimuli, such as prisms and pyramids. Finally, in Experiment 4, we used the most challenging condition of three‐ to six‐angled pyramids at an angle that had their bases concealed from the observer. Working at the edge of extrafoveal processing capabilities, we investigated its plasticity depending on expertise level and individual training. Experiment 4 contained two series with any eye movements away from the screen center prohibited, with training series in between them to reveal the ability of developing covert attention abilities. In Experiment 4, we invited two groups of participants to take part—psychologists as “novices” and mathematicians as “experts”—in order to check if some initially higher‐level perceptual processing could have become automized with the relevant practice. Traditional categorical search studies aim to construct a computational model that would predict group performance in a particular task (Zelinsky et al., 2013). Contrary to this research program, we focused on individual differences and idiosyncratic strategies, which organize the processes of attention and saccade execution during visual search.

## 2 Experiment 1: Extrafoveal processing of simple geometric shapes

### 2.1 Method

#### 2.1.1 Participants

Twenty subjects (12 females, 8 males, aged 18–66) took part in the experiment. All participants reported normal or corrected to normal vision. The sample size in this as well as in all following experiments was chosen based on previous studies investigating categorical search (we used the similar sample sizes as in Alexander, Zelinsky, 2012; Chen, Zelinsky, 2006; Maxfield, Zelinsky, 2012). All experiments were conducted according to the principles of the Declaration of Helsinki and approved by the local ethics committee (Faculty of Psychology, Lomonosov Moscow State University). All participants gave informed consent before the investigation.

#### 2.1.2 Apparatus

We used SMI RED 120 Hz for eye movements fixation and iViewX software for their recording. The stimuli were presented on a 19‐inch monitor with a 60 Hz refresh rate using Experiment Center 3.3.

#### 2.1.3 Stimuli and experimental design

**Fig. 1:**
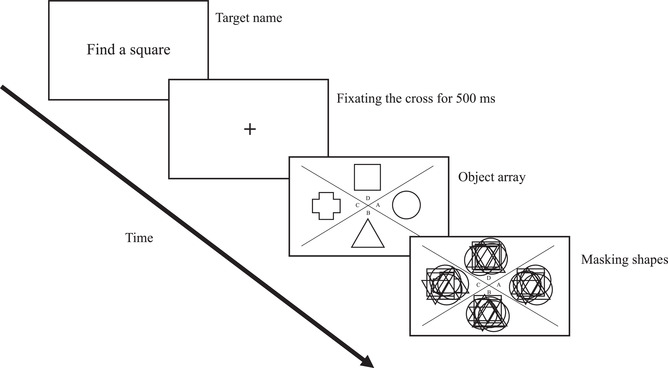
Schema of the frame sequence in a typical trial. The target category name was exposed at the beginning of the trial. After pressing the space button, a fixation cross appeared that needed to be fixated for 500 ms to trigger the presentation of the object array. When the participants found the target, they were to press the space button. After that, the object array was replaced with masking shapes. The participants had to name the letter of the sector with the target shape. Note that all instructions were in Russian; here, we translate them for clarity.

The stimuli comprised simple geometric shapes: a circle, a triangle, a square, and a cross that were positioned at four areas on a screen as illustrated in Fig. 1. Each area was labeled: A, B, C, and D. Shapes had 4–6° size and their centers were located with the eccentricity of 12° of visual angle from the center of the screen; that is, each was located in the extrafoveal visual field. The stimuli were presented on a white background with a viewing distance of 60 cm.

The experiment contained 24 trials grouped by blocks of four with the same target. The target could be a circle, a triangle, or a square depending on the experimental block. The cross was not used as a target since it is a peculiar geometric shape distinct from all the common ones and it is not convex, which is rare for the everyday environment. All combinations of the shape locations were varied, and each target appeared twice in all sectors.

#### 2.1.4 Procedure

Before the experiment, each participant underwent a 12‐point calibration; an accuracy of 0.5° or less was considered as appropriate. Each trial began with the target category name, which the participants were asked to read, then to press the space button and to fixate their gaze at the center of the screen (i.e., the fixational cross; see Fig. 1). After 500 ms of fixating the gaze, stimuli appeared. The task was to find the target as quickly and accurately as possible, press the space button, and pronounce the letter of the target area (A, B, C, or D). After pressing the space button, the shapes were changed to specially drawn masking stimuli with all shapes mixed, preventing recognition based on after‐image processing. The researcher recorded all answers to check their accuracy afterward.

#### 2.1.5 Data processing

**Fig. 2:**
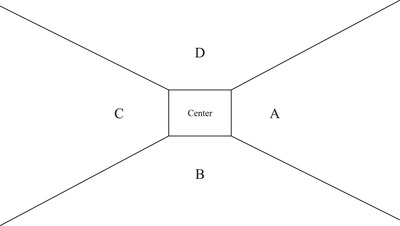
The areas of interest for analysis.

All answers of all participants were correct. The only parameter of our interest was the efficiency of extrafoveal search for the target. It was calculated as follows. Using BeGaze 3.3 software, the screen was divided into five areas of interest (AOI): A, B, C, D, and center (Fig. 2). Then we considered the fixation sequence of the AOI in each trial. The main parameter for all series of the experiment named FirstT (i.e., the FIRST visiting of Target) was the number of the first target sector name appearance in the sequence. The first fixation at the center as well as the repeated fixations at the same sectors was not taken into account thus identifying how many other shapes a participant had gazed at before the target shape was attended. For example, the fixations ran in the following way: “Center, A, B, B, A, C.” This means that if the target is in the C sector, FirstT equals 3 as we do not count the center and the repeated fixations on sectors B and A.

The FirstT parameter was chosen to refer to a predicted mean number of visited sectors under the assumption that the shapes were not processed extrafoveally. If we assume that a participant does not use extrafoveal vision while searching, the target area could be visited first, second, third, or fourth in the stochastic sequence of all visited sectors, and consequently the mean number of the target area visits (FirstT) would be 2.5. Theoretically, a participant does not need to visit the fourth sector when all three previous ones have not revealed the target and could just name the last sector as a target. However, this situation was never observed: in such a situation the participants always visited the target sector and confirmed the target presence before pressing the space button. Unlike the FirstT parameter, the total number of fixations before visiting the target could be unpredictably large, as the participant could resample each shape many times. Thus, introducing the FirstT parameter allowed us to test if extrafoveal analysis had been involved by comparing the FirstT value with 2.5.

Preliminary data processing was performed using Python, and statistical analyses were conducted using IBM SPSS software version 21.

### 2.2 Results and discussion of Experiment 1

The total number of trials in all participants was 24 x 20 = 480 trials. The main focus of our research was the efficacy of extrafoveal processing. We revealed that in 371 trials (77.5% of total amount), the target was found without any eye movements away from the central sector, and in 73 trials (15% of total amount), the target was reached from the very first saccade.

Even though the task turned out to be very easy, we observed pronounced individual differences in the extrafoveal processing data (Crosstable Respondent × FirstT: χ^2^ = 258, df = 76, *p* < .0001: One of the participants gave only three answers without any saccades, and 14 answers from the very first saccade, while three other participants managed to find the target without any eye movements in all trials. These findings reveal that some participants mainly relied on the extrafoveal guess about the target location and answered immediately without eye movements, whereas the others, although correctly choosing the direction of the first saccade, still made this confirming saccade to be sure the target was indeed there. This reflects different search strategies in different participants, which can be observed even in such a simple experiment.

## 3 Experiment 2: Categorical search for 2‐D shapes

In Experiment 1, the simple geometric shapes were easily identified extrafoveally; in Experiment 2, we raised the task difficulty to check whether extrafoveal analysis would change while performing the categorical search for more complicated shapes. We also aimed to observe the influence of the distractor and spatial orientation factors on the search efficacy.

### 3.1 Method

#### 3.1.1 Participants

Thirteen subjects (9 females, 4 males, aged 18–25) with normal or corrected‐to‐normal sight participated in this experiment.

#### 3.1.2 Apparatus, procedure, and data processing

The apparatus, experimental procedure, and data processing were identical to Experiment 1.

#### 3.1.3 Experimental design and stimuli

The experimental design was similar to Experiment 1, yet the number of trials for each participant was 96. We used a 2 × 2 × 3 design with three factors: target‐distractor similarity (whether the distractors were more or less similar to the target), the shapes’ spatial orientation (on its base or at an angle), and target type which was either (1) a square, (2) rectangle, or (3) a square named rectangle—the instruction was to find a rectangle, but the target was a square (being a special case of a rectangle). The trials were grouped into blocks of eight trials with the same target. Each block had all combinations of the factors, and the target sector was varied quasi‐randomly to appear twice in each position.

**Fig. 3:**
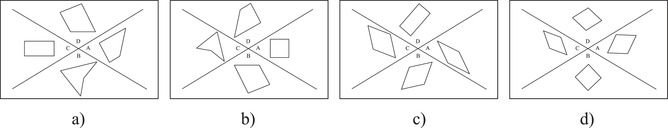
Examples of the stimuli used in Experiment 2: (a) a rectangle on its base among dissimilar distractors; (b) a square on its base among dissimilar distractors; (с) a rectangle at an angle among similar distractors; (d) a square at an angle among similar distractors.

The stimuli comprised both targets and a set of distractors. Squares and rectangles were used as targets. General quadrilaterals were used as dissimilar distractors for both squares and rectangles. Rhombi were used as similar distractors for squares, while parallelograms were used as similar distractors for rectangles (see Fig. 3).

### 3.2 Results and discussion of Experiment 2

We acquired 1241 trials from 13 participants (Table 1); some of the trials (0.56%) were excluded due to technical reasons. We also excluded the trials of Participant K1 due to a high number of mistakes (12.5% vs. 3%–4% in other participants, *SD* = 3.4%). Interestingly, this participant gave the majority of answers without any eye movements, reflecting a dominant guessing strategy that could lead to randomly correct answers and could not be used to infer any deeper about the overall use of foveal processing.

**Table 1:** The number of the first target visit distribution in Experiment 2

Participant ID		The Number of the Target First Visit					Mean FirstT
		0	1	2	3	4	
	А1	77	10	5	0	3	0.34
	A2	31	40	18	4	3	1.04
	A3	20	37	30	6	3	1.32
	D	61	23	9	2	1	0.53
	G	51	33	8	3	1	0.65
	I	31	36	22	6	0	1.03
	K2	11	63	13	9	0	1.21
	K3	36	30	23	5	2	1.36
	L	63	22	7	2	1	0.50
	M1	8	28	29	18	9	1.91
	M2	5	34	38	16	3	1.77
	M3	8	33	33	15	7	1.79
Total		402	389	235	86	33	1.12

Note. FirstT, the FIRST visiting of Target.

We analyzed the results of all participants (except for K1) in all trials and received the mean value of FirstT that equaled 1.12. We also considered individual results and found that all participants showed significant differences in FirstT from the hypothetical mean value 2.5. The individual mean values varied from 1.91 (*t*(91) = –5.04; *p* = .00002 (with four trials spoiled for this participant)) to 0.34 (*t*(95) = –24.9).^1^ An analysis of the results provided in Table 1 shows that 402 of 1145 trials (35%) were solved correctly without any eye movements away from the screen center, and in 389 trials (almost 34%), the very first saccade was directed toward the target. This means that in most cases, the identification task was performed using solely extrafoveal processing. Table 1 clearly demonstrates that the ability of extrafoveal processing and/or strategy of its use vary significantly across the participants (crosstabs participant × FirstT: χ^2^ equals to about 500, df = 48, *p* < .000001). We can also see that the Participants M1, M2, and M3 performed a relatively small number of trials without eye movements away from the screen center, and the mode of their FirstT distributions was 2. On the contrary, Participants A1, В, and L gave answers without any eye movements in more than half of the trials, so the mode of their FirstT distributions is equal to 0. Furthermore, these three subjects had a very small number of incorrect answers (3, 3, and 1, respectively). The latter indicates that they relied mainly on extrafoveal processing.

**Fig. 4:**
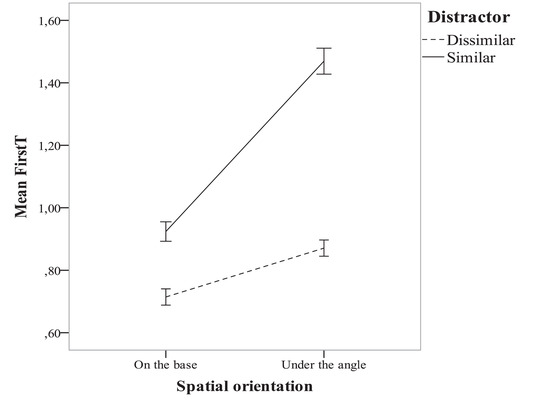
The dependency of mean FIRST visiting of Target (FirstT) from target‐distractor similarity and spatial orientation factors.

This experiment also aimed to check what factors would deteriorate extrafoveal processing and make the participants perform one or more saccades toward the stimuli. In this experiment, we had 3 factors in 3 × 2 × 2 experimental design for the target and distractors: the shape of the target (a rectangle and two kinds of tasks with a square as a target) as the first factor, the spatial orientation of the stimuli as the second factor, and the type of distractors (more or less similar to the target) as the third factor. After conducting Mauchly's sphericity test, we performed a repeated measures three‐way anova. The dependent variable was the mean individual measures for every of 3 × 2 × 2 conditions of FirstT. The independent variables in both analyses were: “Target type,” “Spatial orientation,” and “Distractor type.” There was almost no influence of the “Target type” on FirstT, so we report the dependence of FirstT on the two other factors. Fig. 4 presents the dependency of FirstT from the spatial orientation and distractor type factors.

One can observe the increasing FirstT indicating less extrafoveal processing in the case of an “unusual” spatial orientation as compared to the shapes’ orientation on their bases: the influence of the spatial orientation factor was *F*(1, 11) = 45.1, *p* = .00003, partial *η*² = 0.82. This result might be explained by a prototypical phenomenon in geometrical concepts related to the frame of reference, such as page or screen sides (Hershkowitz, 1998). Another explanation might derive from brain mechanisms of processing horizontal and vertical directions in comparison with oblique directions (B. Li, Peterson, & Freeman, 2003), thus making identification of the right angles in the shapes easier in the case of horizontal bases.

Similar tendencies were revealed for the factor of distractor type, which increased FirstT in the condition of similar‐to‐the‐target distractors as compared to the dissimilar ones: *F*(1, 11) = 38.5, *p* = .00007, partial *η*² = 0.79. The interaction of these two factors was *F*(1, 11) = 10.6, *p* = .008 (Fig. 4), partial *η*² = 0.49. The results of Experiment 2 are largely consistent with the literature data exploring the influence of a distractor on the efficiency of a search (Alexander & Zelinsky, 2011, 2012; Chun & Potter, 1995; Reingold & Glaholt, 2014; Töllner, Conci & Müller, 2015). Particularly, Reingold and Glaholt (2014) demonstrated that the extrafoveal processing of similar distractors required only 24–58 ms, which is too fast to be provided by foveal vision. Therefore, extrafoveal analysis plays the main role in saccade programming and defining whether foveal processing is necessary (p. 629).

The findings of Experiment 2 revealed pronounced individual differences in perception processes and task solving. These individual differences can influence extrafoveal processing, which has already been shown in other works (Frömer et al., 2015; Gandini, Lemaire, & Dufau, 2008), while the current study provides evidence for essential individual differences particulary in the way categorical information guides overt attention. Subjective factors, such as motivation, situation aspects, and personality traits could form and modify the search strategies. They could vary from the extremely “careful” strategy when each extrafoveal guess was checked in the fovea (cases M1, M2, M3) to the “guessing” strategy when the answer was based only on some preliminary extrafoveal analysis (case A1).

Considering the results of Experiments 1 and 2, we can observe a high avaliability of extrafoveal processing, which allows participants to direct a single saccade to the target shape or to identify the target location without any eye movements at all. The factors of distractor type and spatial orientation significantly influenced the first target visit and deteriorated the extrafoveal processing. The results revealed that simple geometric shapes and 2‐D shapes are quite easy to detect extrafoveally, so in the following experiment, we complicated the stimuli to check the limit of extrafoveal processing.

## 4 Experiment 3: Categorical search for 3‐D shapes

We have documented that extrafoveal processing participates in the identification of simple 2‐D geometric shapes differently depending on conditions, but oftentimes such stimuli can be easily detected without any saccades or from the very first saccade. We also aimed to investigate eye movements during the search for more complex three‐dimensional shapes. Here, we intend to find the level of target complexity that makes extrafoveal analysis difficult or even impossible; thus, we varied the factors that we expected would influence difficulty, such as the similarity between the target and distractors, orientation, and type of the target shape. We also hypothesize that individual differences may be expressed in qualitatively different strategies of solving the task with or without extrafoveal analysis in such a complicated case. One additional research question concerns the influence of training on extrafoveal processing. To address this question, we studied the dynamics of extrafoveal processing involvement from the beginning to the end of the experiment in different participants.

### 4.1 Method

#### 4.1.1 Participants

Twelve students and graduates (8 females, 4 males, aged 19–27) with normal or corrected‐to‐normal sight participated in the study.

#### 4.1.2 Design and stimuli

**Fig. 5:**
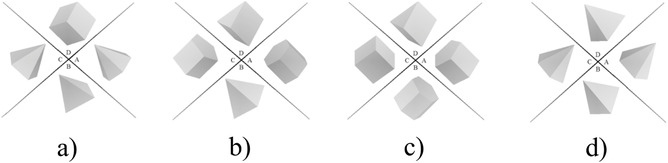
Examples of the stimuli in Experiment 3: (a) a target prism among pyramids; (b) a target pyramid among prisms; (c) a target prism among prisms; (d) a target pyramid among pyramids.

The experiment included 128 trials each comprising four stereometric shapes (regular prisms and pyramids) with three, four, five, or six base angles. Every shape was presented as a projection of a 3‐D scene to the screen plane. The angle between the view axis and the base plane in the 3‐D scene was 35°; the prism/pyramid was quasi‐randomly rotated around the view axis to the angle of ±45° or left unrotated. The target and the distractors could be either a pyramid or a prism. In every trial in which the three‐ to six‐angled shapes were presented, the target could have only four or five angles. The experiment was designed so that the target type could be either the same as or different from the distractor type in half of all trials. Each set of 16 trials were united into series with eight trials in a row containing the target prisms and eight trials in a row with the target pyramids. The target area (A, B, C, or D) was quasi‐randomly varied for each eight trials (see Fig. 5).

#### 4.1.3 Data processing

Given the four different task conditions, we assumed that people would have different strategies in the degree of involvement of extrafoveal processing. So we expected that some respondents would use a strategy close to stochastic foveal viewing of stimuli, while others would use extrafoveal analysis in a different form before planning saccades.

Analyzing diverse strategies that can be based on individual *p*‐values raises a problem of multiple comparisons. When testing many individuals, we would inevitably find some deviations from the stochastic strategy. So we elaborated a special criterion that allows to test the global null hypothesis,^2^ thus demonstrating the non‐occasional occurrence of diverse strategies. This criterion may be called “meta‐criterion of significance distribution” (MCSD). Here, we provide a very short explanation. Let X be the feature inherent to some respondents from the set. The global null hypothesis on the participants’ X is as follows: All participants have a zero level of X. Alternative hypotheses may include the following: (Hypothesis 1) all participants have a (maybe weak) tendency to positive (negative) X level; (Hypothesis 2) only some participants have a definite tendency to positive (negative) X level; and (Hypothesis 3) some participants have positive X level, and others have negative X level. To test all three alternatives, for each participant we calculated *p*‐values of *t* by a one‐sample *t* test, comparing FirstT with 2.5 as we did in the previous experiments. For the global null hypothesis (in this case, it would state that all participants do not use extrafoveal processing at all), the theoretical distribution of *p*‐values would be uniform at (0, 1). To take into account that extreme values on two sides of the *t* test distribution signify opposite individual strategies, we did not analyze *p*‐values of two‐sided tests, but the one‐side *p*‐value (exactly the weight of the left “tail”), which could vary from 0 to 1. Taking together the entire set of *p*‐values, we evaluated the difference between the empirically cumulated *p*‐values distribution function and their theoretical uniform distribution function, using Kolmogorov–Smirnoff (KS) statistics. For the alternative Hypothesis 1, traditional approaches, such as the *t* test, which tests the average of mean FirstTs by subject, would correctly reject the null hypothesis. For the alternative Hypothesis 2, MCSD would be more powerful than the criteria working with the mean or median, since it allows us to detect that some participants have particular (non‐random) strategies, even although these strategies are not universal for populations. For the alternative Hypothesis 3, any criterion working with the mean or median might provide evidence in favor of the null hypothesis while at least some participants have a definite tendency toward a positive X level and some toward a negative one (see an example of such a case in Fig. 11).

Therefore, opposite individual strategies may compensate each other and diminish the *t‐*value, thus putting us at risk of following the null hypothesis when it is not true. On the contrary, MCSD allows us to distinguish whether these opposite strategies are the results of random variability or represent significantly different tendencies.

In the following text, we provide graphs of the *p*‐distribution, illustrating the specific kind of alternative to the H_0_. The difference between the empirical cumulated distribution function, which is represented by circles in the figures below, and the theoretical distribution function, which is represented by a diagonal line, characterizes the difference between the empirical distribution and the uniform one. By evaluating the maximum of this difference, the KS statistic shows the significance of the alternative hypothesis, stating whether the H_0_ may be rejected globally (though not always on average). In some cases, we report on particular subjects who showed results withstanding the Bonferroni correction. By “withstanding the Bonferroni correction,” we mean that the corrected p‐value is less than 0.05 (fully understanding the conditionality of this boundary). Nevertheless, we consider this information to be useful.

### 4.2 Results and discussion of Experiment 3

In this experiment, we analyzed extrafoveal processing in four task conditions representing different combinations of the target and distractors: (1) prism among pyramids; (2) pyramid among prisms; (3) prism among prisms; and (4) pyramid among pyramids. We excluded trials with incorrect answers, without fixations on the target area (0.5%). We also excluded two participants’ data in Conditions 2 and 4 and one participant's data in Condition 3 due to a very high number of incorrect answers (more than two standard deviations). One of the four data sections described below contains the data of 12 participants, one of 11, and two of 10 participants. We acquired 1535 trials for the further analysis.

#### 4.2.1 Target prism among pyramids (Fig. 5a)

**Fig. 6:**
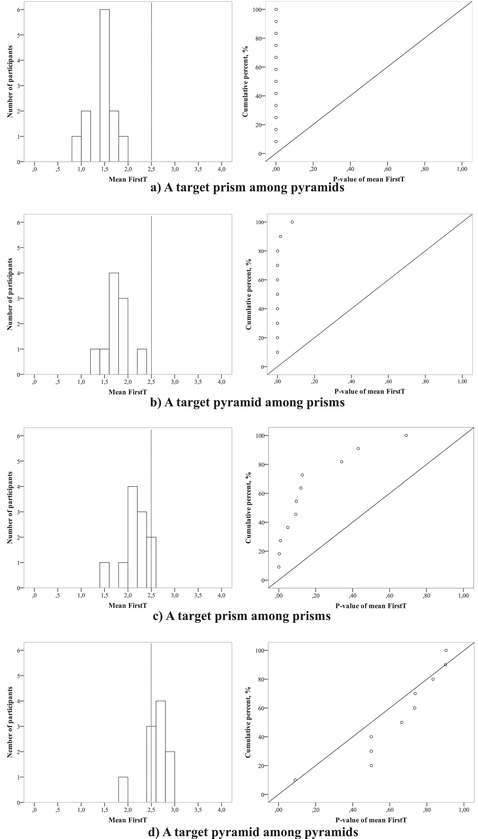
Individual mean FirstT distribution in all participants (on the left) and cumulative distribution of *t* test p‐values of the difference between mean FirstT and 2.5 in each participant (on the right) in the conditions (a) “target prism among pyramids,” (b) “target pyramid among prisms,” (с) “target prism among prisms,” and (d) “target pyramid among pyramids.”

This condition turned out to be the easiest for our participants. The average of individual participants' mean FirstTs was 1.44 (*t*(11) = 11.17, *p* < 0.00001. Individual mean FirstTs varied from 0.97 (*t*(31) = 12.9, *p* < .00001) to 1.91 (*t*(31) = 3.8, *p* = .001), so they were significantly lower than 2.5 (Fig. 6a, on the left), and *p*‐values of the *t‐*statistics did not exceed .001 in any participant (Fig. 6a, on the right). Our meta‐criterion MCSD demonstrated that *D*(12) = 0.99, *p* < .00001. The visualization of MCSD shows the corresponding *p*‐values of the difference between individual mean FirstTs and tested value 2.5; and one can see that in this easy condition, *p*‐values (circles) are all located on the left side near 0 (Fig. 6a, on the right). All participants demonstrated a high involvement of extrafoveal processing, which was reflected in the very first saccade being directed to the target—or even the absence of any saccades—in more than half of the trials. Additionally, only one answer of one participant was incorrect.

#### 4.2.2 Target pyramid among prisms (Fig. 5b)

This condition was also simple for almost all participants, but two of them made multiple mistakes (surprisingly, about a half of the trials within this type), so we excluded them from the analysis. The average of individual mean FirstTs was 1.76 *t*(9) = 9, *p* < .00001, and it varied from 1.38 (*t*(31) = 8.9, *p* < .00001) to 2.24 (*t*(31) = 1.46). Our criterion MCSD showed *D*(10) = 0.92, *p* < .00001 (Fig. 6b, on the right), indicating the apparent involvement of extrafoveal processing while solving this type of task.

**Fig. 7:**
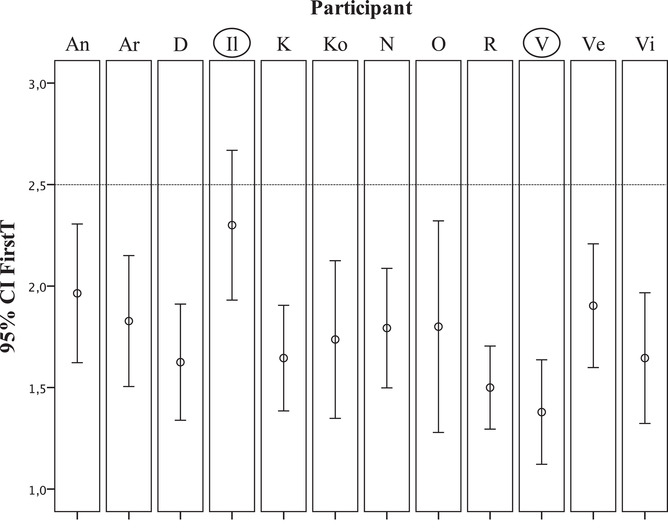
95% confidence intervals (CI) of mean FirstT in all participants in the “target pyramid among prisms” condition. Participants Il and V (in circles) were chosen for the further analysis.

**Fig. 8:**
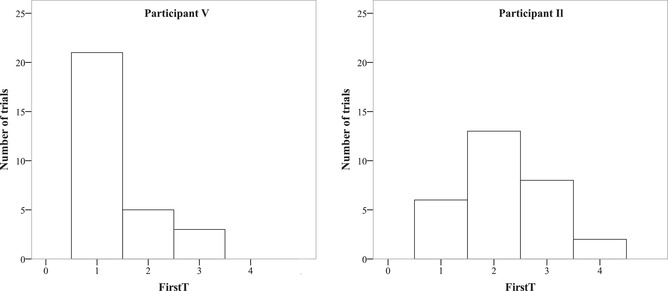
FirstT distribution in trials of the “target pyramid among prisms” condition in Participant V (on the left) and Participant Il (on the right).

The analysis of 95% confidence intervals (CI) for each participant's FirstTs in this condition showed that almost all participants successfully used extrafoveal processing since their CIs did not include 2.5 (Fig. 7). The CI of only one participant (Il) included this critical value, so it is interesting to consider this case as opposed to the case of Participant V as having the most “extrafoveal” strategy. Even in this very easy condition, we can observe two opposite cases in Participants V and Il: the mean FirstT in V was 1.38, and the mode of their distribution was 1 (Fig. 8, on the left), whereas the mean FirstT in Il was 2.24, and the mode of their distribution was 2 (Fig. 8, on the right). So while participant V's first saccade almost always hit the target sector, the target sector was visited first, second, third, or even fourth by Participant Il.

#### 4.2.3 Target prism among prisms (Fig. 5c)

Visibility of the prism bases allowed participants to calculate the number of base angles, so the number of mistakes was very small. Still, this condition turned to be more difficult than the previous two since it used the same type of object for the target and distractors. This difficulty was reflected in a higher average of mean FirstTs by subject equaling 2.19 (*t*(10) = 3.78, *p* = .004), which varied from 1.58 (*t*(30) = 5.15, *p* = .0002) to 2.59 (*t*(31) = –0.5, *p* = .61), meaning that some participants might not have used extrafoveal processing (Fig. 6c, on the left). MCSD showed *D*(11) = 0.546, *p* = .001, evidence for the differences among the participants: Fig. 6c (on the right) clearly shows that while some participants certainly involve extrafoveal vision (three *p*‐values close to 0), others do not (*p*‐values tending to stretch along the diagonal representing the theoretical uniform *p*‐value‐distribution); here, MCSD is more powerful than Student's *t* test.

#### 4.2.4 Target pyramid among pyramids (Fig. 5d)

This condition turned to be so challenging that only two participants made a few mistakes (0 and 4), while the others gave a lot of wrong answers (*M* = 13.8; *SD* = 7.3). Though the further analysis does not have much significance due to the small number of trials with correct answers, still, we repeat the previous logic of presenting results. The average of mean FirstTs by subject exceeded 2.5 (*M* = 2.75; *SD* = 0.14), *t*(9) = 1.36, *p* = .207) and did not improve during the experiment, so it was the most complex condition. The participants’ mean FirstTs varied from 2.08 (*t*(9) = 1.5, *p* = .09 to 2.94 (*t*(17) = –0.14, *p* = .193; Fig. 6d, on the left). The MCSD demonstrated that *D*(10) = 0.44, *p* = .021 (Fig. 6d, on the right). This result indicated the necessity of repeated foveal analysis for some participants.

The findings of Experiment 3 revealed a pronounced influence of the distractor factor on extrafoveal analysis efficiency while searching for three‐dimensional geometric shapes. In the condition with heterogeneous targets and distractors, the order number of the target sector visit was less than 2.5, as it would be in the case of solely foveal analysis. This result points to the presence of an extremely fast parallel processing of all stimuli and the decision to guide overt attention only to a few of the most relevant objects or confined only to extrafoveal analysis. By contrast, in the condition with the homogeneous targets and distractors, we observed a decrease in the efficiency of extrafoveal analysis. In the case of the target prism among prisms, however, extrafoveal processing still contributes to the analysis—perhaps due to the visible bases of the shapes and therefore the number of angles that can be identified without overt attention. Recognizing the target pyramid among the distractor pyramids was much more complicated since their bases were hidden and there were no obvious cues. In this case, the participant has to rely on the mental image of the target shape, as well as its parameters relative to the other stimuli.

We also revealed individual differences in involving extrafoveal processing while solving the search task. The differences were most evident in the condition with the target prisms among distractor prisms, a task of medium complexity. In this condition, some participants obviously relied on extrafoveal processing, whereas others tended to use mostly overt attention (see Fig. 6c). In the most complicated condition with the target pyramid among distractor pyramids, most participants made a lot of fixations and comparisons before answering and scarcely used extrafoveal data. This means that we got close to a limit of extrafoveal analysis capabilities in this task. Further, we conducted a new experiment with the most challenging condition to investigate the limits of extrafoveal processes in depth.

## 5 Experiment 4: Categorical search for pyramids

In the fourth experiment, we investigated in depth the extrafoveal analysis in the most complex condition of the previous trial—the target pyramid among distractor pyramids. We had already found that extrafoveal processing poorly contributes to the analysis of such stimuli, so in this experiment, we intended to understand if and to which degree extrafoveal processes might join the categorical search in this difficult task, depending on a variety of factors. We investigated whether extrafoveal vision could be trained and whether it can contribute to the categorical search in restricted conditions with eye movements prohibited. To study the plasticity of extrafoveal processing and its dynamics across trials in detail, we used four measures: (1) the dynamics of mean FirstT from the beginning to the end of the experiment for each participant, (2) the dynamics of the general time (GenT) of task solving in each trial, (3) the dynamics of the number of correct answers, and (4) performance in the restricted condition with the prohibition of eye movement and its dynamics. We also hypothesized that the relevant educational profile could contribute to more efficient extrafoveal processing of stereometric shapes. Thus, in this experiment, two groups took part: Mathematicians, physicians, and programmers were considered to be “experts” (since they passed challenging mathematic exams including geometry), and psychologists were considered to be “novices.”

### 5.1 Method

#### 5.1.1 Participants

The sample included two groups: 13 “experts” and 16 “novices” (20 females, 9 males, aged 19–28). All participants had normal or corrected‐to‐normal vision.

#### 5.1.2 Design and stimuli

The stimuli involved only the pyramids of the previous experiment, but they were rotated to quasi‐random angles from –45 to 45 degrees (see Fig. 5d). The experiment consisted of nine series of 16 trials. In the first series, participants were introduced to the procedure and it was not taken into account in the analysis. In series 2 and 9, participants were instructed to maintain their gaze at the center of the screen, so any eye movements were prohibited. Series 3–8 were given for training and participants could freely move their eyes. We modified the stimuli in series 2 and 9 in comparison to 3 and 8 to make sure that the training influences categorical search rather than perceptual familiarity with the shapes: The angles between the view axis and base plane were 25° for the training series and 35° for the series with prohibited eye movements.

#### 5.1.3 Data processing

The data processing was identical to what was used in the previous experiments, with the additional analysis for the FirstT and GenT dynamics, which were calculated as the regression coefficients of the FirstT and GenT with the trial order number. We also compared the efficiency of categorical search in the trials with prohibited eye movements before and after the training. In this condition, trials with fixations out of the center sector were excluded from the analysis.

### 5.2 Results and discussion of Experiment 4

There were 4162 trials from 29 participants. The between‐group analysis failed to show any differences between the “experts” and “novices” in the number of correct answers: The first group gave 74.2% correct answers and the second gave 70.1%. The absence of significant differences between the two groups may indicate that this task requires some specific skills that are not directly connected with an educational profile. In post‐experimental interviews, psychology students with better results (more than 80%) discussed their experience with schematic representations of three‐dimensional shapes at school or art/design classes. Since there was no significant difference between the groups, we then analyzed both groups together.

Considering the results of the training sessions 3–8, we compared the average of mean FirstTs in each participant with 2.5, using the one‐sample *t* test, and received 2.28, *t*(28) = 4.9, *p* = .00004. While analyzing mean values, we considered only the trials with correct answers, though one could object that correct answers are given for easier trials. However, if we analyzed all the trials independently of their answer correctness, the results would be even stronger: *t*(28) = 5.3. Therefore, we can state that extrafoveal processing was involved in performing the categorical search in this experiment, despite its similarity with the cases of *target pyramid among pyramids* in the third experiment (paragraph 4 in the results), where extrafoveal processing was not observed. We explain this difference by the fact that in Experiment 3, the trials with *target pyramid among pyramids* were quasi‐randomized with the others. The participants in Experiment 3 did not have a chance to develop an efficient strategy of categorical search that would involve extrafoveal vision since the three other conditions were relatively easy and it could have been challenging to create a separate efficient strategy for the most difficult condition. In Experiment 4, there were many trials with *target pyramid among pyramids* stimuli, so efficient utilization of extrafoveal vision could emerge. This result might speak to the plasticity of extrafoveal processing involvement in categorical search depending on the broader set of surrounding tasks.

**Fig. 9:**
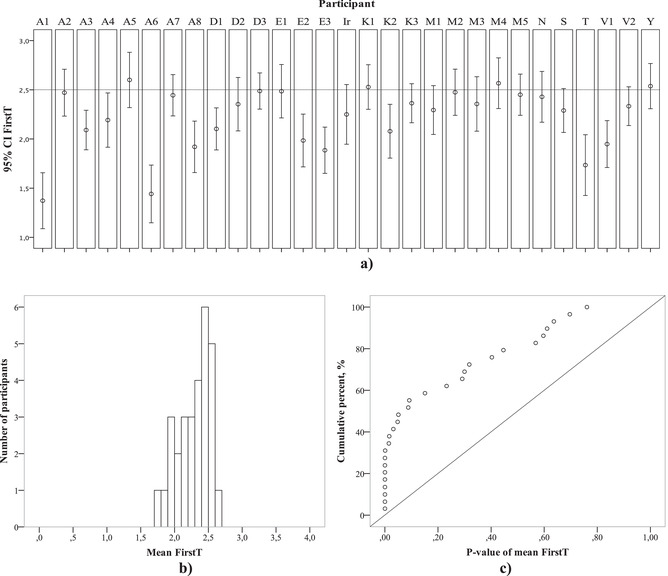
Individual mean FirstT 95% CI (a), individual mean FirstT distribution (b), and *p*‐value cumulative distribution of mean FirstTs (c) in all participants in Experiment 4.

Notwithstanding the apparent mean measures, we also revealed individual differences. The MCSD showed *D*(29) = 0.315, *p* = .005, thus revealing that some participants clearly used extrafoveal vision. The results of the one‐sample *t* test for each participant showed *p* < .001 in seven participants, and it was significant even after Bonferroni multiple comparisons correction for 29 comparisons. The distribution of individual mean FirstTs showed that the distribution mode was located near 2.5 (Fig. 9b), and the CI varied a lot (Fig. 9a). This may be interpreted as corroborating that about half of participants might not have used extrafoveal analysis when planning saccades, while the others clearly did use it (see the points with very low *p*‐values in Fig. 9c).

**Fig. 10:**
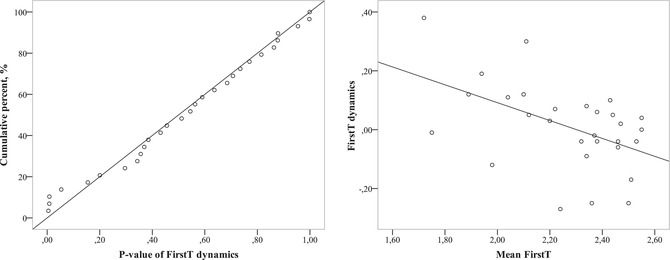
The significance of mean FirstT dynamics across trials (on the left) and scatter plot of individual FirstT dynamic (individual correlation coefficient of FirstT and Trial number) and individual FirstT means (on the right) for 29 participants.

We also analyzed the dynamics of FirstT across the experiment: A negative correlation between FirstT and the trial number would mean a gradually more effective use of extrafoveal analysis during the experiment. The mean of these correlations across the group is equal to 0.0004 (*SD* = 0.16), thus revealing no progress in the use of extrafoveal vision in the sample as a whole. However, there are some participants who clearly demonstrated an increased or decreased use of extrafoveal processing (see Fig. 10, on the left). Including the trials with both correct and incorrect answers, we obtained a noticeable negative correlation—a decrease in the use of extrafoveal processing—for three participants whose correlations were –.271, –.246, –.248 with corresponding *p*‐values .009, .016, .016 (they do not withstand the Bonferroni correction). A noticeable increase of FirstT was observed in two participants (*r* = .307, *p* = .004 and *r* = .375, *p* = .0015; the latter withstands Bonferroni correction for 29 comparisons). For the whole group, a negative correlation between the participant's FirstT dynamics and mean FirstT was observed (see the scatter plot in Fig. 10, on the right). Spearman's correlation coefficient was –.46 (*p* = .011), and Pearson's provided a better result (–.5, *p* = .006). This means that those participants who involved extrafoveal processing more tended to decrease this involvement during the experiment and vice versa. It was Participant T who showed the best mean FirstT had the most pronounced impaired FirstT across the experiment (both significant after Bonferroni correction).

**Fig. 11:**
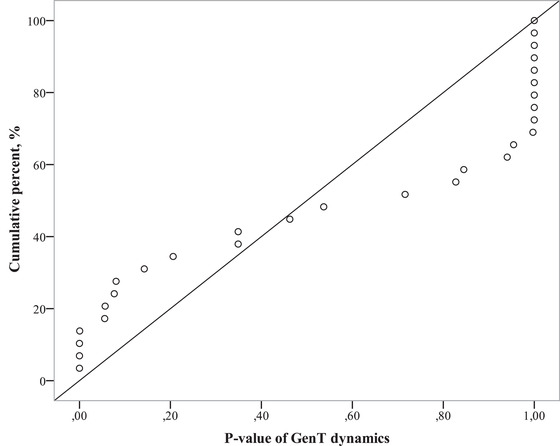
The empirical cumulated distribution function of left‐tail significances of the general time regression coefficient. The left side corresponds to time increasing; the right side to time decreasing. The theoretical function for uniform distribution is shown as a diagonal line.

The dynamics of time paying for each task solving (GenT) are of additional interest. As in the previous case, we measure this dynamic for each participant by GenT and trial number correlation. Fig. 11 represents the empirically cumulated distribution function of these individual correlation significances. The left side corresponds to GenT increasing, the right side corresponds to GenT decreasing. The significance .995 at the X‐axis indicates .005 one‐tail significance of negative regression coefficient for a participant.

The right side of Fig. 11 presents ten participants’ significances that are less than .0015 (right one‐tail, eight of them withstand Bonferroni correction); the left side also presents four participants’ significances less than .0015 (left one‐tail, three of them withstand Bonferroni correction). MCSD is *D*(29) = 0.35, *p* = .0009. The difference between theoretically cumulated distribution functions (diagonal line in the graph) and the empirically cumulated distribution has two extrema (see Fig. 11). This difference has a maximum at the point corresponding to eight participants with the most pronounced GenT decrease (at the point *p* = .9995) as well as a minimum of about .2 at the point *p* = .08.^3^

Thus, we obtained convincing evidence of opposite tendencies in GenT dynamics within the sample, and they may correspond to different strategies for solving the task. The time increasing may indicate not only participants’ fatigue but also their putting more effort into giving a correct answer or a shifting of criterion in the speed‐accuracy trade‐off. The correlation between the regression coefficient for GenT and the logistic regression coefficient of answer correctness is .3, so an increasing time of task solving is connected with increasing the frequency of answering correctly, which is in favor of the criterion shifting interpretation.

**Fig. 12:**
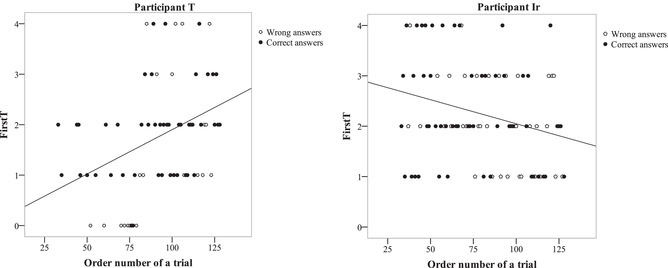
Changing FirstT across trials in Participant T (on the left) and Participant Ir (on the right). Black circles show correct answers and white circles show incorrect answers.

To illustrate the complexity of the dynamics and individual differences, we describe two vividly opposite cases: One participant (T) tended to use extrafoveal processing more actively at the beginning of the experiment and used it less and less toward the end (Fig. 12, on the left), whereas the other participant (Ir) demonstrated an increase of involving extrafoveal processing across trials (Fig 12, on the right).

For Participant T, the mean FirstT in training series 3–8 was 1.79, and the comparison with 2.5 showed *p* < .0001; the regression coefficient of the FirstT to the trial number was .0.38, *p* = .0015 (both *p*‐values withstand Bonferroni correction); and the percentage of correct answers increased across the experiment (the logistic regression coefficient equaled .24, *p* = .003). GenT increased during the training series (the regression coefficient was equal to 31 ms/trial, which corresponds to a 3‐s increase during the training series). This participant chose to solve the task more accurately at the middle of the experiment after a series of very quick unsuccessful guesses with FirstT equal to 0 around trial 75 (see Fig. 12, left).

For Participant Ir, the tendency was precisely the opposite: the mean FirstT was 2.24, *p* < .013; the regression coefficient of the FirstT to the trial number was –.23, *p* = .009; and the percentage of correct answers decreased (the logistic regression coefficient equaled –.17, *p* = .029; Fig. 12, right). It took less and less time for this participant to solve the task (the regression coefficient of the GenT for a trial to the trial number was equal to –8 ms/trial, *p* < .00001; for the entire sequence it was –768 ms/96 trials). This subject seemed to be increasingly less interested in the task and seemed to rely more and more on extrafoveal analysis without attempts to solve the task accurately.

Other participants demonstrated various patterns of dynamics across the experiment. For example, five respondents who revealed a pronounced decrease of time for task solving (more than one was demonstrated by Participant Ir) had stable a FirstT and stable correct answer percentages. Thus, one can see that the involvement of extrafoveal processing and other related variables could change over time due to different reasons, which we plan to investigate in further research on the basis of participant interviews and qualitative analysis.

The most interesting results were obtained for series 2 and 9, in which any eye movements away from the screen сenter were prohibited, and thus categorical search could be performed only by covert attention. There were no differences between mathematicians and psychologists in the ratio of correct answers: 0.56 and 0.57 in the second series and 0.68 and 0.69 in the ninth, respectively. Further statistics are therefore reported for the two groups together. We revealed that participants were able to identify the target shape using only extrafoveal processing, and this ability turned out to improve with practice. There were more correct answers in the ninth series as compared to the second: 0.68 and 0.56, respectively, *t*(28) = 2.71, *p* = .011 (*p* = .027 for non‐parametric test). The comparison of series 2 and 9 shows the forced involvement of extrafoveal processes being trained. Comparing correct answers' frequencies with the random guessing value of 0.25 shows *t*(28) = 7.5 and *t*(28) = 16.4 in the second and ninth series, respectively.

Moreover, taking into consideration an evident similarity between a three‐ and four‐angled pyramid, as well between a five‐ and six‐angled pyramid, and the noticeable difference between these pairs (see Fig. 5d), we also compared the frequency of correct answers with 0.5, reflecting random guessing, after the pair identification. As we already mentioned, in the second series, the mean frequency was 0.56 (*t*(28) = 1.49, *p* = .15), while in the ninth it was 0.68 (*t*(28) = 6.99, *p* < .000001). This result indicates successfully solving a categorical search task by using only covert attention. Note that the correlations between the percent of correct answers in both the second and ninth series and the mean FirstT in each participant were small and equaled .085 and .11, respectively, which means that the potential ability to involve extrafoveal processing, which we observe in categorical search with prohibited eye movements, does not mean participants involve it in ordinary categorical search.

Evidence of training was also shown by the results of logistic regression, with the number of correct answers in a trial being the dependent variable and the trial number being the independent variable. The number of correct answers in a trial increased from series 3 to series 8: the regression coefficient was .008, *p* = .000002 (with the argument varying from 1 to 96); MCSD was *D*(29) = 0.41, *p* = .00007.

To summarize, the results from the series with prohibited eye movements allow us to conclude that (i) extrafoveal processing in the second series with prohibited eye movements is quite effective; (ii) the participants somehow learned extrafoveal processing while performing categorical search; and (iii) even though the participants improved their ability of extrafoveal processing, they did not demonstrate the progress in using it in ordinary trials.

Another important finding of this experiment is the modification of strategies by most of the participants in the course of the experiment, and these strategies changed differently in different participants, as evident in a correlation of the extrafoveal involvement with the dynamics in the change of this parameter. Some subjects demonstrated successful training in categorical search, which was manifested in reducing the number of visited sectors and, consequently, in an improvement of extrafoveal analysis effectiveness. Other participants, who initially distinguished stimuli more successfully and had a higher extrafoveal analysis efficiency measure, demonstrated its deterioration in the course of the experiment. These differences were even more pronounced for the dynamics of GenT in the categorical search. It can also be assumed that such factors as the individual characteristics of participants, their perceptual and cognitive strategies, and also situational motivation influenced search efficiency. These findings are consistent with other works in the field (Gandini et al., 2008; Fromer et al., 2015), allowing us to discuss individual differences in the strategies of visual search, as well as their specific changes across the experiment.

Additionally, it was found that identifying pyramids, representing complex geometric shapes, as well as the level of extrafoveal analysis effectiveness, is not directly connected to the educational profile of the participants. Based on post‐experiment interviews, specific experience with three‐dimensional objects, based on knowledge and skills in the fields of art and architecture, may be more interrelated with success in this task.

We studied the organization of perceptual processing and showed that extrafoveal processing is determined not only by low‐level perceptual features but also by characteristics of a higher level provided by mental categorization operations and possibly by the experience in working with complex geometric stimuli. The influence of top‐down mechanisms on the perception process has been shown in previous works (Rosenholtz, Huang, & Ehinger, 2012). As applied to our study, similar results were obtained by Yang and Zelinsky (2009), who showed the effective categorical search for real‐world objects. Our study adds to these findings the involvement of categorical top‐down processes, both in overt attention and in extrafoveal analysis while processing complex geometric shapes.

## 6 General discussion

The aim of the current study was to examine the capabilities of extrafoveal processing during the categorical search for geometric shapes. More precisely, we aimed (i) to investigate the dependence between the efficacy of extrafoveal processing in categorical search and the degree of stimulus difficulty; (ii) to describe individual search strategies; and (iii) to study the plasticity of the covert attention involvement.

A gradual increase of stimuli difficulty allowed us to investigate how extrafoveal processing changes depending on shape complexity. In this vein, Experiment 1 with simple geometric shapes demonstrated that most of the participants gave correct answers based predominantly on the extrafoveal analysis of all stimuli in parallel. Notably, even for such a simple task, we observed strong individual differences reflected in either more “guessing” or more “careful” strategies in different participants. Experiment 2 involved more difficult shapes such as rectangles and squares and confirmed the influence of two factors on overt attention, in line with other studies: the distractor (e.g., Alexander & Zelinsky, 2011, 2012; Reingold & Glaholt, 2014) and spatial orientation (Gregory & McCloskey, 2010; Rosch, 1975). In our study, these factors were shown to influence extrafoveal processing and categorical search efficacy as well. Experiment 3 involved three‐dimensional shapes and revealed conditions of increasing difficulty with less contribution of extrafoveal processing as evidenced by the increased number of fixated stimuli before reaching the target. This experiment also identified the most challenging condition with the target pyramid amidst distractor pyramids, which was investigated in detail in Experiment 4. In the fourth experiment, we found that the categorical search for pyramids was particularly difficult for all participants: They hardly relied on extrafoveal processing during the search and they performed a lot of fixations on the stimuli before answering. However, the findings in the trials prohibiting any eye movements revealed the plasticity of the perceptual strategies: if overt attention was not accessible, extrafoveal processing was sufficient for categorical search with much higher accuracy than random search.

The present study provides fresh evidence that extrafoveal processing of the whole visual field is involved in the categorical search from the array onset: In many cases, the very first saccade was directed to the target stimulus or participants gave a correct answer without any eye movements. This finding suggests that categorical search relies on the global functioning of pre‐attentive and covert attention mechanisms, as they are distributed across the visual field and provide information relevant for the search from the very beginning of stimuli presentation (Treisman, 2006). One possible explanation is that the observers could access information about the stimuli‐category relations through immediate and parallel processing of definite sets of visual features that mostly define this category (Evans & Treisman, 2005). For example, simple 2‐D shapes like the circle and triangle in Experiment 1 differed greatly due to the presence or absence of angles. A similar effect was shown for more complicated 3‐D shapes like the pyramids and prisms in Experiments 3 and 4: They differed in visible or invisible bases. An alternative explanation might be as follows: A substantial amount of information including higher‐level conceptual data can be preserved in visual form, such as prototypical images (Hershkowitz, 1998) or perceptual symbols (Barsalou, 1999), and is available for comparison with the external data from extrafoveal vision, either pre‐attentively or covertly, before the overt attention deployment. Furthermore, our data could be considered as evidence in favor of a conceptual load on visual attention. This makes a large set of visual features and object‐selective information accessible from peripheral vision prior to saccadic eye movements (Melcher, 2007), meaning that this information can be used to guide saccadic preparation in a top‐down fashion (Moores, Laiti, & Chelazzi, 2003).

In contrast with Brown et al. (1992), we observed evident differences in processing 2‐D and 3‐D shapes as reflected in the increased number of fixated stimuli in the latter condition and therefore a more frequent necessity to involve overt attention to identify the target. This discrepancy can be explained by the more pronounced homogeneity of three‐dimensional stimuli in our study as compared to the cited one. Furthermore, in our experiments, we used more realistic images of shapes as compared to the ones of the mentioned work, which could also contribute to their better perception.

Although the issue of attention mechanisms has been the focus of a continuous debate for decades, most studies have concentrated primarily on the general tendencies of categorical search. This allowed predicting some specific search behaviors common to all participants irrespective of their backgrounds. Still, many nuances were not covered in this mainstream approach, leaving numerous potentially critical factors contributing to extrafoveal processing beyond the scope of the study. Our research focused not only on the average characterization but also on individual differences in perception and attention processing. This required some modifications of the commonly applied methodology to distinguish individual strategies from random variability to make these differences visible and accessible for analysis.

The methodological novelty of this study is its application of global hypothesis testing to evaluate not only the mean measures of the sample but the performance peculiarities in each particular individual, as well as the group characterization “globally,” as opposed to “on average.” The difference is extremely apparent where the participants showed different tendencies throughout the experiment: There is no doubt that some participants used extrafoveal analysis, and it seems that others did not use it (e.g., Experiment 4). This global hypothesis testing allowed us to avoid multiple comparisons. Note that even if the tendency in any participant cannot be accepted as statistically significant after the Bonferroni adjustment, the distribution of individual *p*‐values may verify the presence of (probably opposite) tendencies, since this distribution differs from the uniform distribution presupposed by the global H_0_ hypothesis.

Our hypothesis about more efficient extrafoveal processing in a professional group comprised of “experts” was largely disproved. These “experts” performed just the same as the “novices.” At the same time, post‐experimental interviews bring some evidence that broader relevant experience with three‐dimensional shapes might contribute to a higher involvement of extrafoveal processing. Further investigation of this hypothesis requires studies with other relevant participant groups, such as architects, designers, and so forth.

Having documented that some of the participants used extrafoveal analysis intensively in the ordinary trials, we also took the opportunity to examine the plasticity of extrafoveal processing by introducing special trials prohibiting any eye movements away from the screen center. Other works have already demonstrated that forced covert attention enhanced performance in a texture segmentation task (Yeshurun & Carrasco, 1998), increased spatial resolution (Carrasco, Loula, & Ho, 2006), and heightened contrast sensitivity at the target location (Pestilli & Carrasco, 2005). In line with the previous research, we revealed that forced covert attention provides a significantly higher number of correct answers as compared to random in the most complicated condition with pyramid shapes, even though most participants tended not to use covert attention in ordinary trials. This forced condition presents a fundamental ability to identify even the exceptionally challenging shapes in extrafoveal vision in most participants. Since most participants were able to use it successfully in the forced task, it again highlights the individual strategies used in the free trials with more or less pronounced involvement of extrafoveal processing.

The findings of the current study also suggest that the generation of the first saccade may be quite an automatic process rather than requiring top‐down control. However, in some participants, changing the search strategy during the experiment may indicate a form of some tactical override and strategic delays of saccade execution. Additionally, using the flexible strategy for both easier and more difficult conditions in Experiment 3 prevents a development of extrsfoveal processing in the most difficult condition. Experiment 4 included the most difficult task and demonstrated effective extrafoveal processing in the condition with the prohibition of eye movements as well as in the free trials. This finding is consistent with Findley (1997) who assumed the same tendencies based on unexpectedly short latencies even in challenging conditions.

To sum up, our findings, at least for simple 2‐D geometric shapes, suggest that the target category can be processed in extrafoveal vision very early and plays an important role in guiding overt attention. This result is largely consistent with previous works showing that overt attention guidance is fulfilled not only by low‐level visual saliency (Chen & Zelinsky, 2006). However, the current study demonstrates that when increasing the shapes’ difficulty, the role of extrafoveal processing declines, thus reflecting the necessity for foveal analysis of the objects. Although we suppose categorical characteristics to be more influential as compared to visual features, at least for more complicated shapes, it is still to be determined whether effective categorical identification is based on a parallel detection of low‐level features defined for a category (Evans & Treisman, 2005; Walther & Shen, 2014) or on a holistic perceptual representation of conceptual information critical for the object category (Seidl‐Rathkopf, Turk‐Browne, & Kastner, 2015; VanRullen, 2009; Wyble, Folk, & Potter, 2013). Therefore, further research is needed to more thoroughly investigate which processes are involved in extrafoveal processing in a categorical search task.

## 7 Conclusion

The findings of the present study revealed a significant role of extrafoveal processing in the categorical search for geometric shapes. This was reflected in the smaller number of fixated objects prior to the target selection as compared to a random choice of fixated objects. In the condition with simple geometric shapes and 2‐D shapes, the target was identified from the very first saccade or even without any saccades. With an increase of the shapes’ difficulty, the role of extrafoveal processing declined as reflected in the increased number of fixations at the stimuli, thus indicating the necessity for foveal analysis. Even for simple stimuli, we observed individual differences resulting in various strategies with more or less pronounced degrees of extrafoveal processing. The trials that prohibited eye movements away from the screen center evidenced the principal ability to use covert attention and extrafoveal processing and t efficacy in the most challenging condition with barely distinguishable geometrical shapes. Moreover, we have shown the plasticity of extrafoveal processing, since it can be trained. Further research is necessary to examine the possibilities of extrafoveal processing during a categorical search for other higher‐level stimuli to differentiate bottom‐up and top‐down tendencies in visual perception. Overall, the current study provides evidence of the profound contribution of extrafoveal processing in the complicated process of categorical search.

## Conflict of interest

The authors confirm that there is no conflict of interest in this manuscript.

## Notes

1 We understand that using a *t* test for asymmetric distribution can provide a vulnerable result. Indeed, here we have the right distribution asymmetry that results in an understatement of *p*‐values when the hypothetic mean exceeds the sample mean. Still, we claim that the difference between the observed and hypothetical FirstT is reliable, given that *p*‐values are less than .0001.

2 Some general mathematical details on global testing may be found in Arias‐Castro, Candès, & Plan, 2011 and Candes, 2018.

3 For the minimum, the KS test one‐side *p*‐value is about .05. The simultaneous estimation of both maximum and minimum differences of the distribution function with KS criterion is in our case of opposite tendencies absolutely correct.
